# Simultaneous perfusion and dopaminergic imaging using dual-isotope CZT SPECT/CT in dementia with Lewy bodies

**DOI:** 10.1186/s13550-026-01386-z

**Published:** 2026-02-07

**Authors:** Chawki Gayor, Christian Scheiber, Marc Janier, Dumitru Craciun, Sophie Dautricourt, Virginie Desestret, Antoine Garnier-Crussard, Anthime Flaus

**Affiliations:** 1 Hospices Civils de Lyon, Département de médecine nucléaire, Lyon, France; 2 Université Claude Bernard Lyon 1, Villeurbanne, France; 3 Clinical and Research Memory Centre of Lyon, Department of Geriatric Medicine, Lyon Institute for Aging, Charpennes Hospital, Clinical Research Center Aging-Brain-Frailty, Hospices Civils de Lyon, Villeurbanne, France; 4 Université Claude Bernard Lyon 1, CNRS, INSERM, Centre de Recherche en Neurosciences de Lyon CRNL U1028 UMR5292, Bron, F-69500 France; 5 French Reference Center for Paraneoplastic Neurological Syndromes and Autoimmune Encephalitis, Hospices Civils de Lyon, Lyon, France; 6 MeLiS – UCBL-CNRS UMR 5284 – INSERM U1314, Université Claude Bernard Lyon 1, Lyon, France; 7 Neurocognition and Neuro-Ophthalmology Department, Hôpital Pierre Wertheimer, Hospices Civils de Lyon, Lyon, France

**Keywords:** Cadmium-zinc-telluride, HMPAO, FP-CIT, Synucleinopathy, Neurodegenerative disease

## Abstract

**Background:**

Current diagnostic criteria for dementia with Lewy bodies (DLB) include indicative and supportive biomarkers, some of which are typically assessed separately using dopamine transporter or perfusion imaging. Cadmium-zinc-telluride (CZT) Single Photon Emission Computed Tomography (SPECT) cameras improve simultaneous dual isotope acquisition using [^99m^Tc]-hexamethylpropyleneamine oxime ([^99m^Tc]TcHMPAO) (perfusion) and *N*-(3-Fluoropropyl)-2β-carbomethoxy-3β-(4-[^123^I]iodophenyl)nortropane ([^123^I]FP-CIT; dopamine transporter), allowing the assessment of one indicative and two supportive biomarkers in a single session. This study aimed to describe the results of dual-isotope brain SPECT imaging in a cohort of patients clinically diagnosed with DLB and to compare clinical characteristics, cognition, structural atrophy, and Alzheimer’s disease (AD) biomarkers, between imaging outcomes.

**Results:**

This retrospective single-center study included a total of 56 consecutive patients (mean ± standard deviation age 80.4 ± 7.8 years; 34% females) referred from an expert memory center finally diagnosed as DLB and who underwent a dual-isotope brain SPECT. Based on core clinical features from Mc Keith et al. revised diagnostic criteria, 40 patients (71%) were classified as probable DLB and 16 (29%) as possible DLB. Reduced dopamine transporter uptake (indicative biomarker) was observed in 84% of patients, while cerebral hypoperfusion was found in 100%. Supportive biomarkers such as cingulate island sign and occipital hypoperfusion were present in 29% and 55%, respectively. All 9 patients with normal dopamine transporter imaging had cerebral hypoperfusion and 3 of them presented supportive biomarkers of DLB. No significant association was found between imaging biomarker results and global cognition score, each core clinical features individually, medial temporal lobe atrophy, or CSF biomarkers for AD.

**Conclusion:**

Dual-isotope brain SPECT using CZT frequently identifies dopamine transporter abnormalities in patients with DLB, always in conjunction with perfusion abnormalities. Patients with normal dopamine transporter imaging always present perfusion abnormalities. This imaging approach provides a more comprehensive assessment of indicative and supportive biomarkers in DLB, without increasing scan duration, potentially enhancing diagnostic confidence in clinical practice, particularly in challenging cases.

**Supplementary Information:**

The online version contains supplementary material available at 10.1186/s13550-026-01386-z.

## Background

Dementia with Lewy bodies (DLB) is characterized by the pathological accumulation of aggregated alpha-synuclein within the brain [1, 2]. McKeith et al. proposed revised diagnostic criteria for DLB in 2017 [3]; these include four core clinical features and three indicative biomarkers, the combination of which allows to classify patients as having “possible” or “probable” DLB. Additionally, supportive clinical features and biomarkers that can assist DLB diagnostic evaluation have been defined.

Functional imaging allows to assess some of these indicative and supportive biomarkers. Reduced dopamine transporter uptake in basal ganglia is an indicative biomarker that can be identified using *N*-(3-Fluoropropyl)−2β-carbomethoxy-3β-(4-[^123^I]iodophenyl)nortropane ([^123^I]FP-CIT) single photon emission computed tomography (SPECT). Although it has high diagnostic accuracy for DLB [4], [^123^I]FP-CIT SPECT remains negative in 10–15% of autopsy-confirmed DLB cases [5–7]. Supportive biomarkers in DLB include occipital hypometabolism or hypoperfusion, which can be assessed using [^18^F]fluorodeoxyglucose-positron emission tomography (FDG-PET) to investigate cerebral metabolism [8] or [^99m^Tc] hexamethylpropyleneamine oxime ([^99m^Tc]TcHMPAO) SPECT for cerebral perfusion [9]. Another supportive biomarker is the cingulate island sign (CIS), referring to the relative sparing of the posterior cingulate cortex metabolism [3]; this, however, has only been validated using FDG-PET [10].

Recent technological advancements have led to the introduction of SPECT cameras equipped with cadmium-zinc-telluride (CZT) detectors which provide higher sensitivity and improved energy resolution. These features enhance the possibility to perform concomitant dual-isotope brain SPECT using [^123^I] and [^99m^Tc] allowing the assessment of both dopaminergic and perfusion related biomarkers in a single session [11]. This approach could substantially reduce the burden of multiple imaging sessions for frail patients or those with behavioral disorders, while providing complementary diagnostic information [12]. Although the feasibility of dual-isotope CZT SPECT has been demonstrated in phantom and preclinical studies [11, 13, 14], clinical data, particularly for brain imaging, remain limited; moreover, clinical data in DLB are lacking [15, 16]. Additionally, the X-ray computed tomography (CT) component of hybrid SPECT/CT also allows to evaluate the morphological preservation of medial temporal lobe structures, another supportive DLB biomarker [3].

Therefore, this study aimed to describe the results of dual-isotope brain SPECT/CT using CZT detectors in patients with DLB. Additionally, we compared clinical and paraclinical parameters – including clinical features of DLB, global cognition, brain atrophy, and Alzheimer’s disease (AD) biomarkers – between imaging outcomes.

## Materials and methods

### Population

This retrospective single-center study included all consecutive patients referred at our institution between January 1, 2018, and November 1, 2021, who underwent a dual-isotope brain SPECT/CT, had a final diagnosis of DLB confirmed by experienced dementia specialists including neurologists, geriatricians, or psychiatrists [3]. The exclusion criteria were: insufficient data to determine DLB probability according to revised criteria for DLB diagnosis [3] (absence of a detailed medical report including core clinical features and at least one indicative biomarker result) or a significant abnormality on magnetic resonance imaging (MRI) or CT that may alter the interpretation of dual-isotope brain SPECT/CT (i.e., such as stroke or surgical sequela, presence of brain tumor, or significant white matter lesions).

### Data collection

The data extracted from the patients’ medical records were: demographic data (age, sex), presence/absence of parkinsonism, Rapid Eye Movement (REM) sleep behavior disorder (RBD), visual hallucination, cognitive fluctuations, cognitive impairment assessed using a global cognitive score (Mini-Mental State Examination MMSE, range 0–30 with higher score indicating better cognition), and AD biomarkers in the cerebrospinal fluid (CSF) comprising total tau (t-tau), phosphorylated tau (p-tau), amyloid-β₄₂ (Aβ₄₂), and the Aβ₄₂/Aβ₄₀ ratio if Aβ₄₂ was normal. Patients with amyloid positivity based on decreased Aβ₄₂ or decreased Aβ₄₂/Aβ₄₀ ratio and tau positivity based on elevated p-tau concentrations were classified as AD-positive, using validated cut-offs used in clinical practice.

### Image acquisition

Dual-isotope brain SPECT/CT was performed using a Discovery NM/CT 670 CZT hybrid camera (GE HealthCare^™^, Chicago, IL, USA), equipped with a wide energy high resolution collimator. The energy windows were set at 159 keV (−4%; +5%) for [^123^I] and at 140.5 keV (−7.5%; +5%) for [^99m^Tc] [17, 18]. First, 130 MBq (± 10%) of [^123^I]FP-CIT were administered intravenously. Second, 600 MBq (± 10%) of [^99m^Tc]TcHMPAO were injected intravenously, after 10 min of neurosensory rest in a quiet, darkened room, which was maintained until the time of the acquisition. Injected doses followed current international guidelines and dual-isotope SPECT acquisitions were performed within the recommended post-injection windows for each radiopharmaceutical: 180 min for [^123^I]FP-CIT and 30 min for [^99m^Tc]TcHMPAO [19, 20]. The acquisition protocol consisted of 120 projections over 30 min. Subsequently, the CT imaging was performed according to the following parameters: 120 kV, automatic exposure control with a noise index of 6.5 (80–250 mA), pitch = 0.562, rotation time = 0.8 s, slice thickness = 2.5 mm, and matrix size = 512 × 512.

No scatter correction was performed. For visual analysis, [^99m^Tc]TcHMPAO data were reconstructed using ordered-subset expectation maximization with 5 iterations and 10 subsets, incorporating resolution recovery and CT based attenuation correction. A Hann pre-filter (cut-off frequency 0.9 cm^−1^) and a Gaussian post-filter (2 mm full width at half maximum) were applied. [^123^I]FP-CIT data were reconstructed with identical ordered-subset expectation maximization parameters, with a Butterworth pre-filter (cut-off frequency 0.6 cycles/cm, order = 10). For quantitative analysis, [^99m^Tc]TcHMPAO were reconstructed with identical ordered-subset expectation maximization parameters. The same Hann pre-filter was applied but a Butterworth post-filter (cut-off frequency 0.48 cycles/cm, order = 10) was used. [^123^I]FP-CIT data were reconstructed using ordered-subset expectation maximization with 2 iterations and 10 subsets, the same filter was applied.

### Image analysis

Image analysis was blinded to the clinical data. Dual-isotope SPECT images were assessed both independently and superimposed on CT images. [^99m^Tc]TcHMPAO SPECT images were displayed using the French look-up table (LUT) and windowed to the cerebellum while [^123^I]FP-CIT SPECT images were displayed using GE Cool LUT.

Dual-isotope SPECT/CT images were reviewed by two nuclear medicine physician and classified as normal or abnormal based on visual and additionally on semi-quantitative analysis. In case of disagreement, a consensus was reached. For [¹²³I]FP-CIT SPECT, interpretation followed EANM guidelines [20]. The visual analysis consisted in the evaluation of the left and right caudate nucleus, and putamen. Additionally, semi-quantitative analysis was performed using DatQuant™ (GE Healthcare, Chicago, IL, USA). Results were expressed as specific binding ratios (SBRs) and converted to Z-scores by the age-matched normative distribution. [^99m^Tc]TcHMPAO SPECT was evaluated slice-by-slice. Cerebral hypoperfusion was defined as a visually detectable lower perfusion involving at least one cortical region visible on two consecutive slices. The location of each defect was recorded. Additionally, semi-quantitative analysis was performed using QBrain™ (GE Healthcare, Chicago, IL, USA). Standardized uptake ratios in 26 regions of interest were expressed as Z-scores in comparison to the software’s normal database. Surface renderings were used as adjuncts for visualization. Additionally, the readers specifically assessed the presence of occipital hypoperfusion and CIS. The assessment of CIS on perfusion SPECT used the criteria described for [^18^F]FDG PET [10].

Cortical atrophy was visually assessed in the frontal, temporal, parietal and occipital lobes on the CT part of the SPECT/CT. A lobe was considered affected when showing obvious sulcal widening and cortical thinning greater than expected for age and visible on at least two consecutive slices. Discrepancies between both readers were resolved by consensus. Additionally, atrophy of the medial temporal lobe (MTL) was assessed in each hemisphere using the medial temporal lobe atrophy (MTA) score [21], as preservation of the medial temporal lobe is a supportive biomarker for DLB [3].

### Study endpoints

The primary endpoint was to report the dual-isotope brain SPECT/CT imaging findings in patients with DLB. The secondary endpoints were: (1) comparison of the [^99m^Tc]TcHMPAO SPECT results between patients according to the [^123^I]FP-CIT SPECT result; (2) group comparison of patients’ clinical and paraclinical parameters - including each core clinical feature, DLB probable or possible diagnosis, global cognition score, global and MTA CT-derived atrophy, and AD biomarkers – according to SPECT imaging outcomes.

### Statistical analysis

Continuous variables were expressed as mean ± standard deviation (SD) for normally distributed variables and as median and interquartile range (IQR) for non-normally distributed variables. Categorical variables were reported as counts and percentages. Comparisons were performed using the chi-square test for categorical data, with Fisher’s exact test applied when expected frequencies were < 5. The Mann–Whitney U test was used for continuous data due to the limited sample size. A p-value ≤ 0.05 was considered statistically significant. No correction for multiple comparisons was applied.

## Results

### Characteristics of the population

**Fig. 1:**
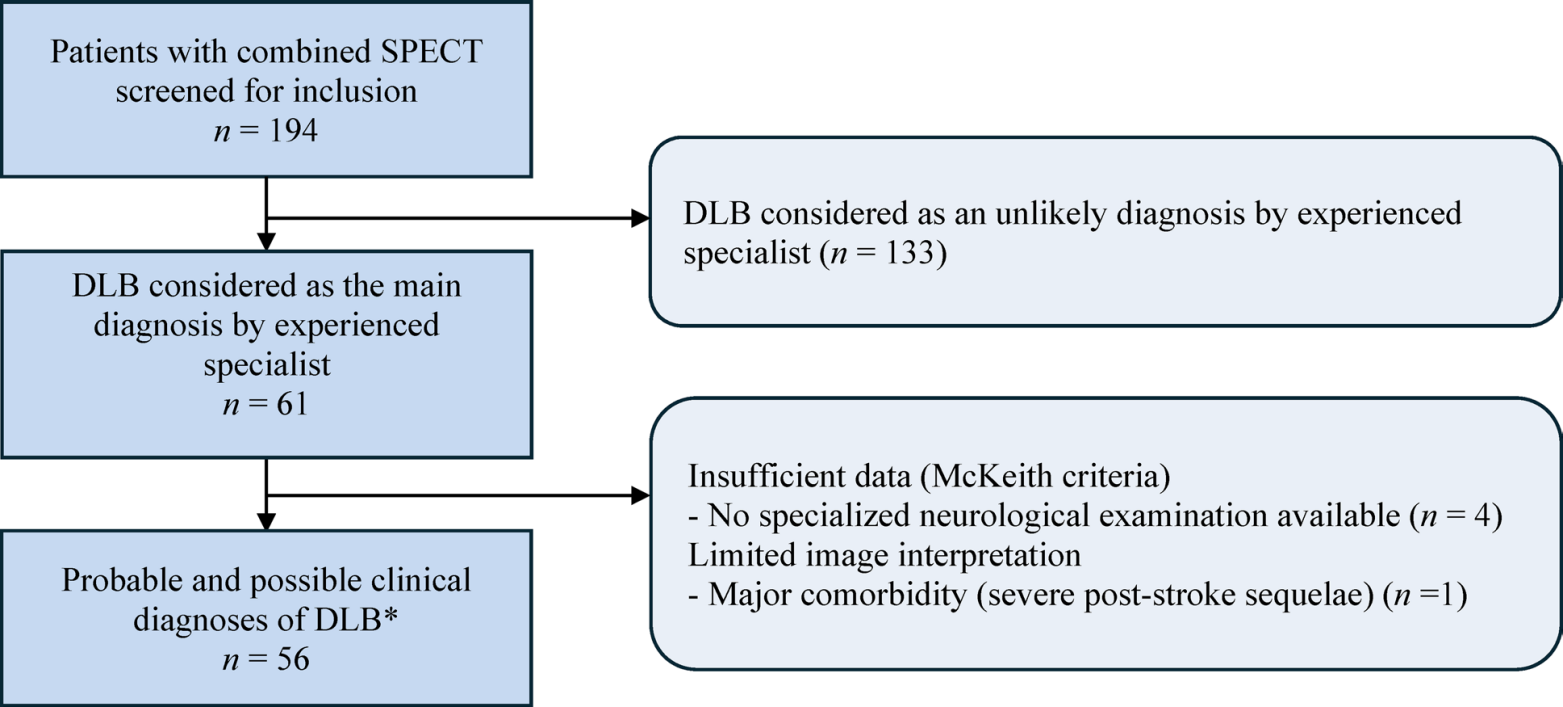
Flow diagram of patient inclusion. SPECT: Single Photon Emission Computed Tomography; DLB: Dementia with Lewy Body; *Clinical diagnosis probability based on core clinical features from revised diagnostic criteria

A total of 56 patients were included (mean ± SD age was 80.4 ± 7.8 years; 34% females; Fig. 1). The median MMSE score was 22 (IQR 19–25). They presented core clinical features: cognitive fluctuations in 29 patients (52%), visual hallucinations in 32 (57%), RBD in 26 (46%), and parkinsonism in 32 (57%). The median time between neuropsychological evaluation (including MMSE) and dual-isotope brain SPECT/CT imaging was 125 days (IQR 50–205). Clinical diagnosis probability according to the core clinical features was probable in 40 patients (71%) and possible in 16 (29%). CSF biomarkers for AD were performed in 20 patients (36%) only and were positive in 6/20 (30%).

### Dual-isotope SPECT findings

**Fig. 2:**
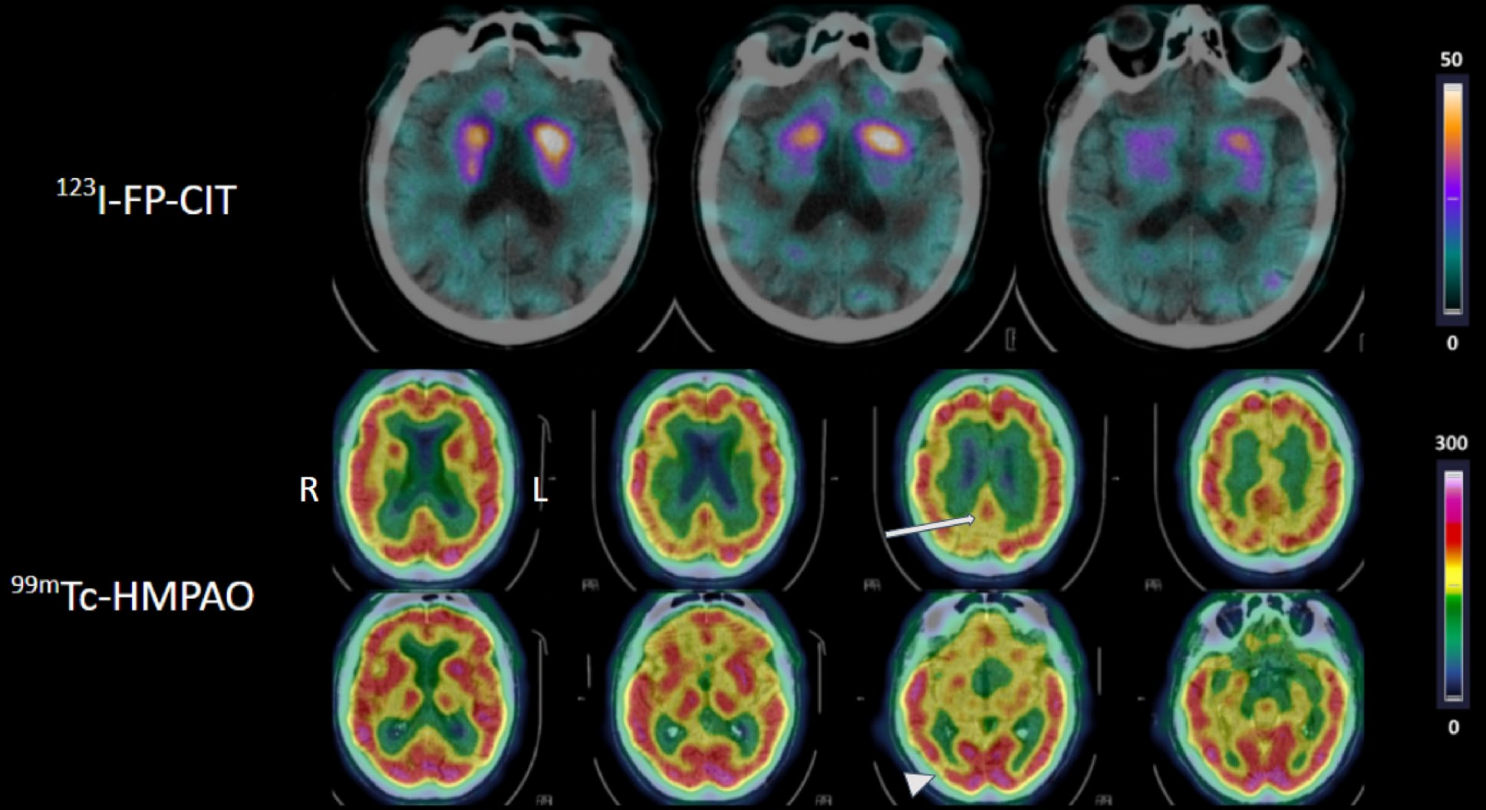
Example of abnormal dual-isotope brain SPECT/CT. The first row displays [^123^I]FP-CIT SPECT/CT axial slices, showing asymmetrical reduced striatal uptake predominantly right. The second and third rows display [^99m^Tc]TcHMPAO SPECT/CT axial slices showing a possible cingulate island sign (arrow) and parieto-occipital hypoperfusion (arrowhead). R: right; L: Left; SPECT: Single Photon Emission Computed Tomography

Dual-isotope brain SPECT/CT (Fig. 2) identified at least one abnormality in all patients (56/56). Reduced dopamine transporter uptake was visually observed in 84% of patients (47/56). The median Z-score derived from SBRs were significantly lower in the group with visually reduced uptake (*p* < 0.0001; Supplementary Table 1). Cerebral hypoperfusion was visually found in all patients (56/56), median values and interquartile range of the Z-score for all regions are given in Supplementary Table 2.

### Brain perfusion imaging supportive biomarkers

A possible CIS was detected in 29% of patients (16/56) but no definite CIS was found. Only the perfusion of the right and left precuneus and the right superior parietal cortex were statistically lower in the group with possible CIS than in the remaining patients. Occipital hypoperfusion was present in 55% of patients (31/56). Perfusion of right and left lateral occipital cortex, right primary visual area, right and left precuneus and right parietal cortex was statistically lower in the group with occipital hypoperfusion than in the remaining patients.

Among patients with possible CIS, normal dopamine transporter uptake was found in 13% (2/16) compared to 18% (7/40) in those without CIS (*p* = 1.0). In case of occipital hypoperfusion, normal dopamine transporter uptake was found in 10% (3/31) compared to 24% (6/25) in patients without occipital hypoperfusion (*p* = 0.16).

In the 9 patients with normal dopamine transporter uptake, supportive biomarkers were present in 3: occipital hypoperfusion only in one patient, possible CIS in another and occipital hypoperfusion and possible CIS in the third.

### SPECT imaging and clinical variables

The MMSE score was not statistically different between patients with positive and negative FP-CIT scans, nor between patients with or without possible CIS, nor between patients with or without occipital hypoperfusion. Likewise, no association was found between these SPECT biomarkers and the presence of individual core clinical features or between the SPECT biomarkers and a clinically probable diagnosis (all *p* > 0.05; Table 1).

**Table 1:** Clinical features according to each imaging biomarker obtained from dual-isotope brain SPECT imaging

	**FP-CIT scan**		**Possible CIS**				**Occipital hypoperfusion**		
	+	-	p-value	+	-	p-value	+	-	p-value
**Number of patients**	47	9		16	40		31	25	
**MMSE score, median [IQR]**	23.0 [18.0–25.0.0.0]	22.0 [21.0–24.0.0.0]	0.96	19.5 [14.8–23.2.8.2]	23.0 [21.0–25.0.0.0]	0.06	23.0 [19.0–24.8.0.8]	22.0 [18.5–26.0.5.0]	0.97
**Symptoms, n (%)**									
** Fluctuations of cognition**	22 (46.8)	7 (77.8)	0.47	8 (50.0)	21 (52.5)	0.87	15 (48.4)	14 (56.0)	0.57
** Visual hallucinations**	24 (51.1)	8 (88.9)	0.27	11 (68.8)	21 (52.5)	0.27	19 (61.3)	13 (52.0)	0.48
** RBD**	21 (44.7)	5 (55.6)	0.72	9 (56.2)	17 (42.5)	0.35	17 (54.8)	9 (36.0)	0.16
** Parkinsonism**	29 (61.7)	3 (33.3)	0.48	10 (62.5)	22 (55.0)	0.61	18 (58.1)	14 (56.0)	0.88
**Clinical probability, n (%)**									
** Probable**	32 (68.1)	8 (88.9)	0.42	12 (75.0)	28 (70.0)	1	24 (77.4)	16 (64.0)	0.27
** Possible**	15 (31.9)	1 (11.1)		4 (25.0)	12 (30.0)		7 (22.6)	9 (36.0)	
**Mesial temporal lobe atrophy**	23 (48.9)	4 (44.4)	1	7 (43.8)	20 (50.0)	0.67	15 (48.4)	12 (48.0)	1

CIS, Cingulate island sign, DLB, Dementia with Lewy bodies; FP-CIT, N-(3-Fluoropropyl)−2β-carbomethoxy-3β-(4-[¹²³I]iodophenyl)nortropane; IQR, interquartile range, MMSE, Mini-Mental State Examination; RBD, REM, Rapid-eve movement sleep behavior disorder; Clinical probability, clinical diagnosis probability according to the revised diagnostic criteria for DLB (based on core clinical features only)

p-values according to chi-square test or Fisher’s test or Mann-Whitney U test

### SPECT imaging and atrophy on CT

Cortical atrophy was observed in 77% of patients (43/56) and in particular MTA in 48% (24/56). The MMSE score was not statistically different between patients with or without MTL atrophy. No association was found between MTA and the three SPECT biomarkers (all *p* > 0.05; Table 1).

### SPECT imaging and CSF alzheimer biomarkers

CSF AD biomarkers were positive in 30% (6/20) of the tested patients; the corresponding SPECT imaging findings are detailed in Supplementary Table 3. Cortical atrophy was observed in 83% of patients (5/6) and in particular MTA in 33% (2/6). The prevalence of occipital hypoperfusion and CIS did not differ between AD-positive and AD-negative cases (5/6 83.3% vs. 6/14 42.9% and 3/6 50% vs. 2/14 14.3%, respectively), and neither did [^123^I]FP-CIT positivity (5/6 83.3% vs. 13/14 92.9%; all *p* > 0.05).

## Discussion

Dual-isotope brain SPECT/CT found an imaging abnormality in all patients of the present cohort with DLB. Reduced dopamine transporter uptake was present in 84% of patients, whereas cerebral hypoperfusion was present in all patients.

Occipital hypoperfusion was observed more frequently in this study than in studies using NaI-based SPECT systems [22, 23], which may partly be explained by differences in detector technology, and suggests a advantage of CZT SPECT [24]. In contrast, for [^123^I]FP-CIT SPECT, a previous study has reported no diagnostic difference between CZT and NaI cameras [25]. Moreover, post-harmonization methods for SBRs have been developed to improve quantitative comparability across camera types [26], further contributing to the robustness of dopamine transporter imaging regardless of detector technology [26]. In our cohort, the proportion of abnormal [^123^I]FP-CIT SPECT aligned with that found in a phase-3 study [4] and in an autopsy-validated series [7]. This similarity can be attributed to the advanced stage of neurodegeneration in these patients, resulting in a markedly reduced uptake that is detectable by both camera types.

Several factors may explain the absence of statistically significant associations in this exploratory study between SPECT results and cognition, clinical features, MTA, or CSF biomarkers. Methodologically, this could be due to the small sample size that limits the statistical power and to the use of a global cognitive score, the MMSE [27], which is not optimal to capture attentional, executive or visuospatial deficits typical of DLB, particularly in case of fluctuations. In addition, prior studies reported heterogeneous or discordant relationships between functional imaging and clinical features in DLB. Reduced dopamine transporter uptake had inconsistent associations with global cognition [28, 29], occipital hypoperfusion did not reliably correlate with visual hallucination frequency [23], and the cingulate island sign showed complex and variable relationships with MMSE, MTA, and clinical symptoms [30, 31]. Additionally, functional alterations often precede detectable structural atrophy [32], which may further explain the lack of association with MTA. Finally, the lack of a significant difference in parkinsonism prevalence between patients with positive and negative FP-CIT is an interesting finding. Two previous studies reported a similar 1:3 ratio of FP-CIT–negative patients presenting with parkinsonism [33, 34]. None of the FP-CIT–negative patients in this study were taking medications known to induce parkinsonism or had extensive vascular abnormalities on MRI.

Among the six patients with available AD-positive CSF biomarkers in this cohort of patients diagnosed with LBD, five showed reduced striatal dopamine transporter uptake and the single patient with a normal dopamine transported uptake exhibited both perfusion supportive biomarker. Because these SPECT findings were suggestive of Lewy body disease, it is plausible that some of these AD-positive cases represent mixed DLB–AD pathology. Such co-pathology is increasingly recognized and is thought to be substantially underdiagnosed in clinical practice [35, 36]. Additionally, four of the six patients showed an absence of MTA on CT, another supportive DLB biomarker [3]. Of course, given the small and non-random nature of the CSF subgroup, the hypothesis that dual-isotope SPECT/CT imaging in conjunction with AD biomarkers could be instrumental in pointing out mixed dementia, will need further confirmation.

Dual-isotope SPECT/CT imaging provides several advantages in clinical practice. By combining both [^99m^Tc]TcHMPAO and [^123^I]FP-CIT acquisition into a single 30-minute examination, it reduces patient burden compared with sequential imaging protocols. This approach is particularly beneficial for older patients who may have difficulty remaining still due to the presence of behavioral or psychological symptoms of dementia [12]. We suggest that this “one-stop” protocol can be useful as an adjunct in challenging diagnoses or atypical presentations; all [^123^I]FP-CIT negative patients still displayed cortical hypoperfusion, and one-third met supportive perfusion criteria for DLB. Overall, dual-isotope imaging may offer valuable insights into atypical parkinsonism and mixed-pathology cases, facilitating a more tailored diagnostic and therapeutic approach [37–39].

Similar approaches using dual-phase PET imaging have been investigated, in which early-phase acquisition serves as a surrogate for cerebral perfusion, and late-phase acquisition reflects dopaminergic transporter binding. Studies using the ^18^F-labelled FP-CIT or the (E)-N-(3-iodoprop-2-enyl)−2β-carbofluoroethoxy-3β-(4’-methyl-phenyl) nortropane ([^18^F]FE-PE2I) PET have demonstrated that both perfusion and dopaminergic information can be obtained from a single radiotracer injection [40–43]. These studies concluded that dual-phase PET acquisition protocols provide diagnostic accuracy similar to separate perfusion and dopamine transporter images, while reducing radiation exposure using a single radiotracer injection within a single day. Additionally, early phase acquisition was proved as accurate as brain FDG PET for differential diagnosis of atypical Parkinsonism [40, 44]. These PET studies highlighted an alternative strategy for obtaining both perfusion-related and dopaminergic information, while illustrating a conceptually related approach to dual-isotope CZT SPECT/CT imaging.

The present study has several limitations. It was conducted in a single tertiary memory center, which may have introduced a selection bias as patients referred to such centers often present with diagnostically complex or atypical cases. However, this setting also reflects real-world clinical practice, in which SPECT/CT imaging is used to resolve diagnostic uncertainty. The relatively high proportion of complex cases may have overestimated the apparent added value of perfusion SPECT. The single center design allowed us to have a consistent imaging protocol and centralized image interpretation, which enhances validity, but may not be reflective of more heterogeneous practice. Another limitation is the assessment of cortical atrophy, which was performed only visually on CT images acquired concurrently to SPECT images. Although lobar atrophy was defined using reproducible visual criteria, sulcal widening and cortical thinning on at least two consecutive slices, this approach remains qualitative and reader-dependent. The absence of volumetric quantification may have reduced sensitivity to cortical changes. The absence of a significant association between cortical atrophy and cerebral hypoperfusion should be interpreted cautiously in this context. Furthermore, CT-guided spatial normalization methods, shown to improve striatal quantification in hybrid SPECT/CT, were not implemented here and could have strengthened semi-quantitative accuracy [45]. Nevertheless, this evaluation reflects routine clinical conditions, and was consistent with the real-world design of our study. Since the present analysis was retrospective, some final clinical diagnoses were established based on input from the SPECT findings, which could introduce a level of circularity between the imaging and diagnostic classification. However, all patients were classified retrospectively as clinically probable or clinically possible DLB according to the McKeith criteria core clinical features [3]. In addition, the retrospective design introduced potential confounding factors regarding disease characterization. Since AD biomarkers were not always available, our ability to assess the frequency of AD co-pathology was limited. No patient in the present study underwent autopsy and neuropathological assessment, the gold standard for definitive diagnosis [46]. Nevertheless, the revised diagnostic criteria for DLB were validated in prospective studies [47], and our findings reflect the reality of clinical practice.

Prospective multicenter trials should quantify the incremental diagnostic yield and cost-effectiveness of dual-isotope SPECT/CT using CZT compared to sequential imaging, ideally integrating other in vivo biomarkers such as α-synuclein seed amplification assays in CSF [48] and AD biomarkers in CSF. Further studies should also focus on the identification of the best target population for dual-isotope SPECT, by clearly defining which patients are most likely to benefit from this combined imaging approach.

## Conclusion

The present study demonstrates that dual-isotope brain SPECT using CZT frequently identifies dopamine transporter abnormalities in patients with DLB, always in conjunction with perfusion abnormalities. Even when dopaminergic imaging is normal, perfusion abnormalities are found. This imaging approach provides a more comprehensive assessment of indicative and supportive biomarkers in DLB, without increasing scan duration, potentially enhancing diagnostic confidence in clinical practice, particularly in diagnostically challenging cases.

## Supplementary Information

Supplementary Material 1

## Data Availability

The datasets generated and/or analyzed during the current study are not publicly available due to health data regulation but are available from the corresponding author on reasonable request.

## Abbreviations

AD: Alzheimer’s disease
CIS: Cingulate island sign
CSF: Cerebro spinal fluid
CZT: Cadmium-zinc-telluride
CT: Computed tomography
DLB: Dementia with lewy bodies
FDG: Fluorodeoxyglucose
FE-PE2I: (E)-N-(3-iodoprop-2-enyl)-2β-carbofluoroethoxy-3β-(4’-methyl-phenyl) nortropane
FP-CIT: N-(3-Fluoropropyl)-2β-carbomethoxy-3β-(4-[^123^I]iodophenyl)nortropane
HMPAO: Hexamethylpropyleneamine oxime
IQR: Interquartile range
LUT: Look-up table
MMSE: Mini mental state examination
MTA: Medial temporal lobe atrophy
MTL: Medial temporal lobe
NaI: Sodium Iodide
PCC: Posterior cingulate cortex
PET: Positron emission tomography
REM: Rapid-eye-movement
RBD: REM sleep behavioral disorder
SBR: Specific binding ratio
SPECT: Single photon emission computed tomography
