# Supplementary material for: Simultaneous perfusion and dopaminergic imaging using dual-isotope CZT SPECT/CT in dementia with Lewy bodies
Source: EJNMMI Res. 2026 Feb 7;16:40. doi: 10.1186/s13550-026-01386-z (PMC12972381; doi:10.1186/s13550-026-01386-z)
Supplement: Supplementary file 1 — Supplementary Material 1 [file 13550_2026_1386_MOESM1_ESM.docx]

**Supplementary data**

Supplementary Table 1. Median and interquartile range of Z-scores of specific binding ratios in striatal nuclei according to visual analysis of FP-CIT scan

|  | Visually normal FP-CIT scan | | Visually anormal FP-CIT scan | P-value |
| --- | --- | --- | --- | --- |
| Striatum | | 2.3 [2.0-2.54] | 1.0 [0.81-1.29] | 3.48E-06 |
| Putamen | | 2.2 [1.78-2.36] | 0.9 [0.74-1.12] | 4.31E-06 |
| Caudate | | 2.4 [2.41-2.76] | 1.3 [1.02-1.73] | 6.59E-06 |

FP-CIT, N-(3-Fluoropropyl)-2β-carbomethoxy-3β-(4-[¹²³I]iodophenyl) nortropane

p-values according to Mann-Whitney U test

|  | Median Z score | IQR |
| --- | --- | --- |
| Prefrontal Lateral R | -4.08 | -5.02--2.88 |
| Prefrontal Lateral L | -4.04 | -5.07--2.72 |
| Prefrontal Medial R | -2.84 | -3.9--1.64 |
| Prefrontal Medial L | -2.49 | -3.27--1.55 |
| Sensorimotor R | -0.89 | -2.25--0.02 |
| Sensorimotor L | -0.57 | -1.59-0.53 |
| Anterior cingulate R | -2.42 | -3.31--0.95 |
| Anterior cingulate L | -1.97 | -3.49--1.35 |
| Posterior Cingulate R | 0.06 | -0.88-0.78 |
| Posterior Cingulate L | -0.02 | -1.05-0.74 |
| Precuneus R | -0.35 | -1.65-0.92 |
| Precuneus L | 0.2 | -0.58-1.12 |
| Parietal Superior R | -1.29 | -1.87--0.43 |
| Parietal Superior L | -0.33 | -0.96-0.72 |
| Parietal Inferior R | -4.14 | -5.07--2.87 |
| Parietal Inferior L | -2.99 | -3.97--1.66 |
| Occipital Lateral R | 1.74 | 0.31-3.13 |
| Occipital Lateral L | 2.47 | 1.2-4.27 |
| Primary Visual R | 1.69 | 0.47-2.41 |
| Primary Visual L | 2.09 | 0.8-3.64 |
| Temporal Lateral R | -1.91 | -2.96--0.91 |
| Temporal Lateral L | -1.42 | -2.58--0.47 |
| Temporal Mesial R | -0.34 | -1.39-0.45 |
| Temporal Mesial L | 0.02 | -0.83-0.71 |
| Cerebellum | 1.6 | 1.14-2.15 |
| Pons | -0.13 | -0.88-0.7 |

Supplementary Table 2. Median and interquartile range of Z-scores of standardized uptake ratios across 26 cortical regions of interest on [^99m^Tc]TcHMPAO SPECT

Supplementary Table 3. SPECT imaging findings in patients with positive Alzheimer’s disease biomarkers

| ID | Abnormal dopamine transporter uptake | Occipital hypoperfusion | Possible CIS | Medial temporal lobe atrophy |
| --- | --- | --- | --- | --- |
| 1 | + | + | + | - |
| 2 | + | + | - | + |
| 3 | + | + | + | - |
| 4 | + | - | - | - |
| 5 | + | + | - | - |
| 6 | - | + | + | + |

CIS : cingulate island sign
